# Weight misperception and psychological symptoms from adolescence to young adulthood: longitudinal study of an ethnically diverse UK cohort

**DOI:** 10.1186/s12889-020-08823-1

**Published:** 2020-05-18

**Authors:** Christelle Elia, Alexis Karamanos, Maria João Silva, Maeve O’Connor, Yao Lu, Alexandru Dregan, Peiyuan Huang, Majella O’Keeffe, J. Kennedy Cruickshank, Elli Z. Enayat, Aidan Cassidy, Oarabile R. Molaodi, Maria Maynard, Seeromanie Harding

**Affiliations:** 1 Department of Nutritional Sciences, School of Life Course Sciences, Faculty of Life Sciences & Medicine, King’s College London, Franklin Wilkins Building, London, SE1 9NH UK; 2 ESRC International Centre for Life Course Studies in Society and Health, Department of Epidemiology and Health, University College London, London, WC1 6BT UK; 3 Institute of Psychiatry, Psychology and Neuroscience, Faculty of Life Sciences & Medicine, King’s College London, Denmark Hill Campus, London, SE5 9RJ UK; 4 Department of Population Health Sciences, School of Population Health & Environmental Sciences, Faculty of Life Sciences & Medicine, King’s College London, Addison House, Guy’s Campus, London, SE11UL UK; 5 MRC/CSO Social and Public Health Sciences Unit, University of Glasgow, Glasgow, UK; 6 School of Clinical & Applied Sciences, Leeds Beckett University, CL 413 Calverley Building, City Campus, Leeds, LS1 3HE UK

**Keywords:** Body image, Weight misperception, Nutrition, Psychological symptoms, Ethnicity, Adolescence, Longitudinal study

## Abstract

**Background:**

To evaluate the association between weight misperception and psychological symptoms in the Determinants of young Adults Social well-being and Health (DASH) longitudinal study.

**Methods:**

A longitudinal sample of 3227 adolescents, in 49 secondary schools in London, aged 11–16 years participated in 2002/2003 and were followed up in 2005/2006. A sub-sample (*N* = 595) was followed up again at ages 21–23 years in 2012/2013. An index of weight misperception was derived from weight perception and measured weight. Psychological well- being was measured using the Strengths and Difficulties Questionnaire at 11–16 years and the General Health Questionnaire at 21–23 years. Associations with weight misperception was assessed using regression models, adjusted for socio-economic and lifestyle factors.

**Results:**

White British males and females were more likely than ethnic minority peers to report accurate perceptions of measured weight. At 11-13y, 46% females and 38% males did not have an accurate perception of their measured weight. The comparable figures at 14-16y were 42 and 40%. Compared with male adolescents, more females perceived themselves as overweight or were unsure of their weight but measured normal weight, and this was more pronounced among Indians, Pakistanis and Bangladeshis. At 14-16y, more males perceived themselves as underweight but measured normal weight, and this was more pronounced among Indians. Compared with those who had an accurate perception of their normal weight, a higher likelihood of probable clinically-relevant psychological symptoms was observed among those who measured normal weight but perceived themselves to be underweight (females Odds Ratio (OR) = 1.87 95% CI 1.03–3.40; males OR = 2.34 95% CI 1.47–3.71), overweight (females only OR = 2.06 95% CI 1.10–3.87), or unsure of their weight (males only OR = 1.61 95% CI 1.04–2.49). Among females, the association was driven by internalising rather than externalising symptoms. An accurate perception of overweight was associated with higher psychological symptoms in adolescence and early 20s. Ethnic specific effects were not evident.

**Conclusion:**

Weight misperception may be an important determinant of psychological symptoms in young people, with an accurate perception of normal weight status being protective. Culturally targeted interventions should be considered to promote healthy perceptions of body image.

## Background

The importance of mental health in adolescence has gained recent recognition with international commitment to prevention and timely access to care [1]. Mental health problems in children and young people in the United Kingdom (UK) are common with about 1 in 10 children affected, and with ethnic minority children having lower rates of mental ill health than their British counterparts [2]. Around 50% of mental illness in adult life starts before the age of 15 and 75% by the age of 18 [3]. There is increasing recognition of the association between mental health disorders and physical ill-health, and an abundance of evidence concerning its impact on educational opportunities, work prospects and risk behaviours [4, 5]. In addition, mental health problems in children and young people incur significant financial costs to health, social and other services such as schools and the criminal justice system. In the UK, estimated annual costs per child range between £11,030 and £59,130, with lifetime costs due to conduct disorder being £5.2 billion [6]. Cost of crime attributable to adults who had conduct problems in childhood is estimated at £60 billion a year in England and Wales, of which £22.5 billion a year is attributable to conduct disorder and £37.5 billion a year to sub-threshold conduct disorder.

Several studies have shown a non-linear relationship between mental illness and body mass index (BMI) in adulthood. Findings from these studies suggested higher rates of mental health problems among underweight and obese individuals, but several other studies have shown little or no association [7, 8]. The evidence on whether overweight/obesity is associated with increased psychological symptoms in adolescence is inconclusive [9]. A potential mediating variable is body satisfaction, an attitudinal component of body image, which denotes an investment in and concern with appearance [10]. Whilst body dissatisfaction is strongly related to excess weight, regardless of age, gender and ethnicity [11, 12], it is not consistently associated with underweight which suggests that other factors may influence mental illness in this group [13].

Adolescence represents a critical stage in the development of positive or negative body image [14]. Rapid changes during adolescence in shape and weight due to puberty interact with socio-cultural contexts to influence body image perceptions [14]. Weight misperception, a perceptual aspect of body image relating to over- or under-estimation of weight, is a separate construct from body dissatisfaction [15]. It is, however, unclear whether weight misperception is more common in females than males adolescents and vice versa [16–18]. Salient influences include unrealistic and idealised images of body size in print media, television and social media, cultural ideals and beliefs about body size, and identity development [14]. Despite the associations observed between body dissatisfaction and increased psychological symptoms in adolescence and adulthood in the US and Australia, the implications of body dissatisfaction and weight misperception on adolescent psychological symptoms has received limited attention in the UK and among ethnic minorities [19, 20].

Most studies around the world that focus on ethnic differences in weight misperception and increased psychological symptoms are among African American girls, among whom overweight is associated with greater body image satisfaction than their White American peers [21, 22]. In the UK, ethnic differences in overweight in adolescence are well known. Black Caribbean and Black African girls are more likely to be overweight than their White British peers [23]. One UK study reported ethnic differences in weight control behaviours, with Bangladeshi and mixed ethnicity boys and Pakistani girls reporting more dieting behaviours than their White British counterparts [19]. Another study reported no ethnic variations in the accuracy of self-assessment of weight status [20]. An ethnic minority mental health advantage has been observed for UK ethnic minority adolescents, which contrasts with the high rates of mental ill-health in adulthood [24]. For example, adult Black Caribbeans and Bangladeshis are more likely to be diagnosed with severe mental illnesses as opposed to lower psychological symptoms in adolescence despite more social adversity [20, 25].

The complexity of the UK context warrants in-depth investigation as to whether, despite this resilience in adolescence, the misperception of weight status affects their psychological symptoms.

The overall aim of this study is to evaluate the longitudinal association between weight misperception and psychological symptoms in a multi-ethnic community sample of adolescents with objective and subjective measures of body size. To our knowledge this prospective study is the first UK study to evaluate the association between body weight misperception of body size and psychological symptoms in young people.

## Methods

### Study design

The Determinants of young Adults Social well-being and Health longitudinal study (DASH) sample was recruited between 2002 and 2003 from 51 schools in 10 London boroughs. Details of the study are described elsewhere [26]. A total of 6631 students, aged 11-13y, took part in the baseline survey. The sample was recruited from schools in the London boroughs of Brent, Croydon, Hackney, Hammersmith & Fulham, Haringey, Lambeth, Newham, Southwark, Waltham Forest and Wandsworth. These boroughs were selected as they have high proportions and numbers of people from ethnic minority groups. Schools with at least 5% of people of Black Caribbean descent were identified using school censuses provided by the Department of Education and Skills [26]. Within each borough, schools were selected to enable representation at, above and below the national averages for academic performance based on reports from the Office for Standards in Education.^18^ The classes were randomly selected and were all mixed ability classes. In 2005–06 4777 (88% of children in 49 schools, 72% of the cohort), aged 14-16y, took part in the first follow-up, with the mean follow-up time of 2.62 years (standard deviation 0.22). Two schools did not participate in the follow-up study, one due to space restrictions during building renovations and another due to the pressures of examination timetables [26].

In 2012/13, 10% subsample (*N* = 665, 97% participation rate) took part in the pilot follow-up. The subsample consisted of 107 White British, 102 Black Caribbean, 132 Black African, 99 Indian, 111 Bangladeshi or Pakistani and 115 other (mainly mixed) ethnicities and was sampled to be representative by gender and socio-economic circumstances (SEC) across the 10 boroughs and 51 schools.

### Measures

#### Psychological symptoms

Externalizing (conduct and hyperactivity) symptoms and internalising (emotional and peer-relationship) symptoms as well as a Total Difficulties Score (TDS- a composite latent variable measuring externalizing and internalizing psychological symptoms) were derived from the 20-item self-completed Strengths and Difficulties Questionnaire (SDQ), which is a validated screening tool for ages 4–16 years [27]. SDQ scores were used as continuous outcome variables. Higher scores indicate greater psychological symptoms. The self-report version of the SDQ correlates well with teacher/parent-report versions and clinician-rated assessments and has been validated internationally [28, 29]. In addition, binary variables using cut-offs of TDS >  17, externalizing > 11 and internalizing > 9 were used to identify probable clinical cases of psychological symptoms, based on validation approach in national data where approximately 10% of adolescents had scores within this band.^21^ The General Health Questionnaire (GHQ-12) was used as a continuous measure at 21-23y to assess psychological symptoms (GHQ-12 is as a one-dimensional latent variable which assesses social dysfunction, anxiety and loss of confidence) in adulthood [30, 31].

### Weight misperception

Misperception of body size was assessed using the question *“Given your height and weight would you say you are...”*, with the choice of four response categories 1) About right 2) Too heavy 3) Too light and 4) Not Sure. Measured body mass index (BMI) was derived from the height and weight measures taken by trained survey assistants and calculated as weight (kg)/height(m^2^). In adolescence, participants were classified as underweight, normal weight, overweight or obese based on the 1990 British age and gender specific growth reference curves; normal weight (between − 1 and + 1 SD BMI for age and sex), overweight/obese category (> + 1SD BMI for overweight)/ > + 2SD BMI for obese).^23^ At 21-23y, overweight was classified if BMI was 25–29.9 kg/m^2^, obesity if BMI was > 30 kg/m^2^. Hereafter, perceived weight is denoted by ‘*p.’,* measured weight by ‘m.’, and overweight or obese is referred to as ‘overweight’. Based on comparison of the two sets of data, participants were classified as: [1] *p.normal weight*/m.normal weight [2]; *p.overweight/*m.overweight [3]; *p.normal weight/*m.overweight [4]; *p.overweight/* m.normal weight [5]; *p.underweight/*m.normal weight [6]; *p.unsure/*m.normal weight [7]; *p.unsure/*m.overweight. Another category was derived (*p.underweight/*m.overweight) but was not included as it contained only three participants. At 21–23, due to the smaller sample size, categories 4 and 5 were aggregated as *p.discordant*/m. normal weight and categories 6 and 7 were aggregated as *p.discordant*/m. overweight.

### Covariates

Socio-demographic variables included age (continuous year), gender (male vs female), and ethnicity. Ethnicity was self-defined and checked against reported parental ethnicity and grandparents’ country of birth. Pupils selected their ethnicity from a list of ethnic groups based on the England and Wales Census 2001 categories (White British, Black African, Black Caribbean, Indian and Pakistani/Bangladeshi). Lifestyle factors included smoking (yes/no); alcohol consumption (yes/no), physical activity, based on 37 vigorous sporting activities (e.g. running, cycling, football, kick-boxing) and the frequency of taking part in each activity (every day, most days, weekly, less than weekly, and never) [23], was classified into the number of activities taken per week and coded into five categories: ‘≥5 times/week’, ‘3–4 times/week’, ‘twice/week’, ‘once/week’, and ‘none’; special diets including vegetarianism, religious prohibition of food and slimming diets (yes/no); weight related anxiety was derived from two questions regarding participant’s weight gain worry *(“At the moment … Are you worried about putting on weight?”)* and overeating worry *(“At the moment … Do you feel unhappy if you eat too much?”)*. Family factors included parental overweight (yes/no), and parenting style using the eight-item Parental Bonding Instrument (PBI) [32]. The PBI was used to derive a measure of parental care (warmth; support) and control (discipline; supervision). Scores were recoded into tertiles (1 = low, 2 = medium, 3 = high care/control), based on the distribution of care and of control of the entire DASH follow-up sample. Social adversity was measured using perceived racism at home/ at school/ on the street (yes/no), and the Family Affluence scale (FAS), a measure of individual SEC in adolescence [33]. The FAS is based on the number of family cars/vans, computers, and holidays, and having their own bedroom, categorised as ‘high (≥3)’, ‘medium [1, 2]’ and ‘low (0)’ FAS. Own education to degree level (yes/no) was used as an SEC measure at 21-23y.

### Analysis sample and statistical analysis

Out of 4777 adolescents who participated in both 2002/2 and 2005/2006, 3286 were retained in the sample as anthropometric measurements were obtained for only White British, Black Africans, Black Caribbeans, Indians, Pakistanis and Bangladeshis. Longitudinal analysis was conducted in a sample of 3227 participants aged 11–16 years after excluding missing information on SDQ (*N* = 59). Cross-sectional analysis was conducted on 595 participants aged 21–23 years who had complete data on the GHQ.

Data analyses were conducted with STATA 13.0 (Stata Corp., College Station, TX, USA). Missing data in each categorical variable were recoded as ‘not stated’. A three-level linear regression model with random intercept was used to explore the association between weight misperception and mean TDS, externalizing and internalizing symptoms across adolescence, as there were repeated measures (Level 1) which were obtained from the same pupil (Level 2) at 11–13 years and 14–16 years, respectively, with pupils clustered within 49 schools (Level 3). All variables were considered as time (age)-dependent except gender and ethnicity.

As data used in the main analysis (11–16 years) were collected at two timepoints (2002–03 and 2005–06), the effect of age fitted as a quadratic or cubic function could not be tested. Models included the linear effect of age (grand-mean centered, in years). The continuous measures of TDS, externalizing and internalizing symptoms were initially regressed on weight misperception and age only (Model 1), and adjustments were sequentially undertaken with each variable added singly. Families of models are presented. Model 2 refers to additional adjustments for ethnicity. Model 3 refers to additional adjustments for own lifestyles (current smoking, current alcohol consumption, special diets, weight gain anxiety, and physical activity). The full model (Model 4) refers to additional adjustments for family factors (maternal overweight, paternal overweight, parental care, and parental control) and social adversity (family affluence, racism). The association between weight misperception and probable clinical cases (SDQ TDS > 17, SDQ externalizing difficulties score > 11 and SDQ internalizing difficulties score > 9) across adolescence were examined using the three-level mixed-effects logistic regression with random intercepts. Results for Models 1 and 4 are presented for both continuous and binary SDQ. Results for Models 2 and 3 are presented in supplementary Tables 3 and 4. The model building approach for analysis of mean SDQ was also used for analysis of SDQ cut-offs and mean GHQ (with fewer variables age, gender, ethnicity, education). All models for the analysis based on 11–16-year olds were stratified by gender as there was a significant gender x weight misperception.

## Results

### Occurrence of weight misperception as a function of age, gender and ethnicity

Table 1 (females) and Table 2 (males) show the distributions of the composite variable of weight perception and measured weight at 11-13y and 14-16y. At 11-13y, 46% females and 38% males did not have an accurate perception of their measured weight. The comparable figures at 14-16y were 42 and 40%. Most of these adolescents misperceived a measured normal weight. More females than males perceived themselves as overweight or were unsure of their weight but measured normal weight across adolescence. At 14-16y, more males perceived themselves as underweight but measured normal weight compared with females. Supplementary Tables 1a and 1b (see Additional file 1) show the corresponding ethnic distributions with additional correlates. Across all ethnic and gender groups, the most commonly occurring group was *p.normal weight*/m.normal weight, 38% at 11-13y and 44% at 14-16y.

**Table 1:** Females sample characteristics from 11 to 13 years to 14–16 years - N (%). The Determinants young Adults Social well-being and Health study

	All (***N*** = 1493)		White British (***N*** = 383)		Black Caribbean (***N*** = 351)		Black African (***N*** = 446)		Indian (***N*** = 172)		Pakistani/Bangladeshi (***N*** = 141)	
	11–13 yrs	14–16 yrs	11–13 yrs	14-16 yrs	11–13 yrs	14–16 yrs	11–13 yrs	14–16 yrs	11–13 yrs	14-16 yrs	11–13 yrs	14–16 yrs
Total Difficulties Score (mean ± SD)	10.1 ± 4.8	10.8 ± 4.9	10.2 ± 4.8	11.3* ± 4.9	10.5 ± 4.9	10.9 ± 4.9	10.2 ± 4.8	10.7 ± 4.8	8.8 ± 4.3^	9.6**^±4.5	10.6±5.1	11.4±5.0
Externalising difficulties score	5.1±2.9	6.0*±3.3	5.2 ±2.9	6.3* ±3.6	5.6 ±3.0	6.3*±3.1	5.2±2.9	6.0*±3.2	4.1^±2.4	5.1*^±2.9	4.8±2.3	5.8*±2.9
Internalising difficulties score	5.0±3.1	4.8±2.9	5.0±3.1	5.0±2.8	4.9±2.9	4.6±2.8	5.0±3.2	4.7±3.0	4.7±2.9	4.5±2.7	5.8±3.2^	5.6±3.0
TDS > 17	136 (9)	176 (12)	33 (9)	55 (14)*	36 (10)	42 (12)	41 (9)	47 (10)	8 (5)	12 (7)	18 (13)	20 (14)
Externalising difficulties score > 11	76 (5)	167 (11)*	15 (4)	50 (13)*	17 (5)	29 (9)^*	15 (3)	38 (8)^*	3 (2)	5 (3)^	6 (4)	10 (7)^
Internalising difficulties score > 9	189 (13)	149 (10)	43 (11)	37 (10)	41 (12)	29 (8)	56 (13)	44 (10)	22 (13)	18 (10)	27 (19)	21 (15)
P. normal weight - M. normal weight§	508 (34)	591 (40)	160 (42)	165 (43)	112 (32)^	141 (40)	138 (31)^	173 (39)	55 (32)	66 (38)	43 (31)	46 (33)
P. overweight - M. overweight	152 (10)	176 (12)	33 (9)	32 (8)	46 (13)	52 (15)	50 (11)	65 (15)^	16 (9)	15 (9)	7 (5)	12 (9)
P. normal weight - M. overweight	139 (9)	66 (4)	26 (7)	9 (2)*	50 (14)^	25 (7)^	46 (10)^	23 (5)	11 (6)	5 (3)	6 (4)	4 (3)
P. overweight - M. normal weight	68 (5)	98 (7)	24 (6)	34 (9)	7 (2)^	17 (5)	17 (4)	21 (5)	9 (5)	12 (7)	11 (8)	14 (10)
P. underweight - M. normal weight	117 (8)	111 (7)	32 (8)	22 (6)	26 (7)	23 (7)	31 (7)	31 (7)	12 (7)	18 (10)	16 (11)	17 (12)
P. unsure - M. normal weight	246 (16)	260 (17)	65 (17)	66 (17)	38 (11)	50 (14)	64 (14)	71 (16)^	43 (25)	38 (22)	36 (26)	35 (25)
P. unsure - M. overweight/obese	115 (8)	101 (7)	19 (5)	16 (4)	36 (10)	24 (7)	33 (7)	44 (10)	15 (9)	11 (6)	12 (9)	6 (4)

§ Percentages do not add up to 100 due to missing values

P. is for “perception” - M. is for “measured”

*P < 0.05compared with 11–13 years; ^*P* < 0·05 compared with White British, † compared with females

**Table 2:** Males sample characteristics from 11 to 13 years to 14–16 years - N (%). The Determinants young Adults Social well-being and Health study

	All (***N***=1734)		White British (***N***=484)		Black Caribbean (***N*** = 344)		Black African (***N*** = 372)		Indian (***N*** = 224)		Pakistani/Bangladeshi (***N*** = 310)	
	11–13 yrs	14–16 yrs	11–13 yrs	14-16 yrs	11–13 yrs	14–16 yrs	11–13 yrs	14–16 yrs	11–13 yrs	14-16 yrs	11–13 yrs	14-16 yrs
Total Difficulties Score (mean ± SD)	9.7± 4.7	9.7*± 4.7	10.3 ± 4.5	10.3±4.6	9.7±4.6	9.5*± 4.6	8.7^ ±4.6	8.9*^± 4.5	10.0^±5.4	9.8^±5.4	9.3^ ±4.7	9.6^±4.6
Externalising difficulties score	5.3±3.0	4.4 (3.0)	5.9±2.8	6.6*±3.4	5.7±3.0	6.1*±3.3	4.8^±2.8	5.7*^±3.3	5.0^±3.0	5.9*^±3.4*	4.6^ ±2.9	5.8^*±3.3
Internalising difficulties score	4.4±3.0	3.6±2.6	4.4±3.1	3.7*±2.7	4.0±2.8	3.3*±2.5	3.9^±2.7	3.2*^±2.5	5.0±3.4	3.9*±3.4	4.7±3.3	3.8*±2.5
TDS> 17	212 (12)	144 (8)*†	72 (15)	46 (10)	43 (13)	28 (8)	30 (8)^	25 (7)†	35 (16)	24 (11)	32 (10)	20 (6)†
Externalising difficulties score > 11	76 (4)	167 (10)*	29 (6)	62 (13)*	18 (5)	32 (9)*	10 (3)	27 (7)*	8 (4)	22 (10)*	11 (3)	24 (8)*
Internalising difficulties score > 9	144 (8)	73 (4)*	45 (9)	23 (5)*	19 (5)	14 (4)	20 (5)	11 (3)	30 (13)	14 (6)*	30 (10)	11 (4)*
P. normal weight - M. normal weight	711 (41)†	820 (47)†	215 (44)	239 (50)	149 (43)	165 (48)	143 (38)	179 (48)^*	77 (34)	92 (41)	127 (41)	145 (47)
P. overweight- M. overweight	130 (8)	129 (7)†	45 (9)	44 (10)	20 (6)	26 (8)	17 (5)	16 (4)^	22 (10)	17 (8)	26 (8)	26 (8)
P. normal weight - M. overweight	142 (8)	117 (7)†	34 (7)	19 (4)	33 (10)	31 (9)^	40 (11)	30 (8)	16 (7)	11 (5)	19 (6)	26 (8)
P. overweight - M. normal weight	35 (2)†	28 (2)†	11 (2)	11 (3)	4 (1)	4 (1)	6 (2)	5 (1)	8 (4)	3 (1)	6 (2)	5 (2)
P. underweight - M. normal weight	151 (9)	210 (12)†	38 (8)	48 (10)	18 (5)	35 (10)	34 (9)	53 (14)	31 (14)	37 (17)	30 (10)	37 (12)
P. unsure - M. normal weight	225 (13)†	246 (14)	61 (13)	54 (11)	31 (9)	51 (15)	51 (14)	52 (14)	37 (17)	40 (18)	45 (15)	49 (16)
P. unsure - M. overweight/obese	111 (6)	81 (5)	33 (7)	21 (4)	26 (8)	13 (4)	17 (5)	19 (5)	13 (6)	15 (7)	22 (7)	13 (4)

§ Percentages do not add up to 100 due to missing values

P. is for “perception” - M. is for “measured”

*P < 0.05compared with 11–13 years; ^P < 0·05 compared with White British, † compared with females

Gender, age and ethnic differences in the type of weight misperception were evident. For example, in the first DASH wave, more males than females were classified as *p.normal weight*/m.normal weight, and more females as *p.overweight*/m.normal weight. In the second DASH wave, more males were classified as *p.normal weight*/m.overweight, and more females as *p.overweight/*m.overweight. Overweight was more common among Black Caribbean and Black African males and females throughout adolescence. Black Caribbean females were more likely than White British females to be classified as *p.normal weight*/m.overweight throughout adolescence. In the second wave of DASH study, Black African males and females were generally less likely than their White British peers to be classified as *p.overweight/*m.overweight.

Supplementary Tables 1a and 1b (see Additional file 1) show that weight gain anxiety was common across all ethnic groups at ages 11–16 years. The prevalence was lower among males than females, among whom it increased. Overall, among females, 49% at 11–13 years and 57% at 14–16 years reported weight gain anxiety. The comparable figures for males were 34 and 26%, respectively. Generally compared with their White British peers, ethnic minorities (except for Black Caribbeans) reported more special diets, parental control and racism, and less smoking and alcohol consumption. Black Caribbeans were less likely to be in the high affluence FAS category throughout adolescence.

### Weight misperception and mean TDS, externalizing and internalizing symptoms

Table 3 shows gender specific associations across age between weight misperception and mean TDS, externalizing and internalizing symptoms. For both genders, two categories were independent longitudinal correlates of SDQ TDS in both females and males (Full models); *p.overweight*/m.overweight and *p.underweight*/m.normal weight, were associated with an increase in mean TDS (higher psychological symptoms). Gender differences were observed for two other categories; *p.overweight*/m.normal weight among females and *p.unsure*/m.normal weight among males were independently associated with an increase in mean TDS (Full model). For females, associations between *p.overweight*/m.normal and *p.underweight*/m.normal were driven mainly by higher internalizing symptoms.

**Table 3:** Females and Males: The association between weight misperception and mean SDQ total, externalising and internalising scores from 11 to 13 years to 14–16 years

	SDQ total difficulties score				SDQ externalising difficulties score				SDQ internalising difficulties score			
	B (95% CI)	P > |z|	B (95% CI)	P > |z|	B (95% CI)	P > |z|	B (95% CI)	P > |z|	B (95% CI)	P > |z|	B (95% CI)	P > |z|
	Model 1		Model 4		Model 1		Model 4		Model 1		Model 4	
**Females**												
Fixed Effects												
*Weight perception (*vs. *P. normal weight - M. normal weight)*												
P. overweight- M. overweight	1.98 (1.32, 2.66)	< 0.001	1.03 (0.42,1.64)	< 0.001	0.8 (0.38, 1.21)	< 0.001	0.17 (−0.24, 0.59)	0.42	1.10 (0.70, 1.50)	< 0.001	0.58 (0.18, 0.99)	0.005
P. normal - M. overweight	− 0.11 (− 0.86, 0.64)	0.777	− 0.31(− 0.99,0.37)	0.369	− 0.05 (− 0.52, 0.41)	0.819	− 0.35 (− 0.84, 0.14)	0.17	0.05 (− 0.40, 0.50)	0.818	− 0.19 (− 0.68, 0.28)	0.428
P. overweight- M. normal weight	1.93 (1.14, 2.71)	< 0.001	1.08 (0.35,1.81)	0.004	0.59 (0.11, 1.08)	0.017	0.08 (− 0.44, 0.60)	0.77	1.37 (0.89, 1.84)	< 0.001	1.18 (0.66, 1.70)	< 0.001
P. underweight- M. normal weight	1.40 (1.32, 1.65)	< 0.001	1.24 (0.60,1.88)	< 0.001	0.33 (− 0.10, 0.77)	0.138	0.50 (0.05, 0.95)	0.03	1.06 (0.63, 1.49)	< 0.001	1.31 (0.87, 1.75)	< 0.001
P. unsure- M. normal weight	0.81 (0.31, 1.30)	0.001	0.44(−0.01,0.90)	0.055	0.39 (0.08, 0.70)	0.013	0.01 (−0.31, 0.34)	0.94	0.51 (0.21, 0.81)	0.001	0.40 (0.09, 0.72)	0.013
P. unsure- M. overweight	−0.11 (− 0.83, 0.62)	0.771	− 0.27(− 0.93,0.40)	0.431	− 0.22 (− 0.68, 0.23)	0.334	− 0.25 (− 0.73, 0.24)	0.32	0.29 (−0.15, 0.72)	0.202	0.19 (−0.28, 0.66)	0.424
Random effects												
Level 3 (School) intercept variance	0.38 (0.13, 1.09)		0.09 (0.00, 1.05)		0.17 (0.06, 0.44)		0.05 (0.00, 0.33)		0.09 (0.02, 0.34)		0.05 (0.00, 0.32)	
Level 2 (Child) intercept variance	11.24 (9.89, 12.78)		9.10 (7.98, 10.37)		4.37 (3.86, 4.97)		3.25 (2.81, 3.75)		3.73 (3.26, 4.28)		3.14 (2.72, 2.63)	
Leve l1 (Occasion) intercept variance	12.72 (11.79, 13.72)		11.78 (10.96, 12.67)		4.89 (4.53, 5.28)		4.464 (4.30, 5.01)		4.84 (4.49, 5.22)		4.75 (4.41, 5.12)	
**Males**												
Fixed Effects												
*Weight perception (*vs. *P. normal weight - M. normal weight)*												
P. overweight- M. overweight	2.18 (1.50, 2.87)	< 0.001	1.56 (0.88, 2.24)	< 0.001	0.56 (0.12, 1.00)	0.013	0.44 (0.00, 0.88)	0.05	1.61 (1.21, 2.01)	< 0.001	1.12 (0.72, 1.52)	< 0.001
P. normal - M. overweight	−0.45 (−1.09, 0.18)	0.162	−0.43 (−1.05, 0.18)	0.164	−0.37 (−0.77, 0.04)	0.076	−0.28 (− 0.68, 0.12)	0.16	0.01 (− 0.35, 0.38)	0.948	− 0.06 (0.43, 0.30)	0.733
P. overweight- M. normal weight	1.60 (0.41, 2.78)	0.008	0.73 (−0.41, 1.88)	0.208	0.78 (0.02, 1.53)	0.045	0.44 (−0.30, 1.18)	0.24	0.72 (0.02, 1.42)	0.043	0.22 (−0.47, 0.90)	0.534
P. underweight- M. normal weight	1.05 (0.51, 1.60)	< 0.001	0.99 (0.47, 1.52)	< 0.001	0.41 (0.06, 0.76)	0.021	0.44 (0.07, 0.75)	0.02	0.63 (0.32, 0.96)	< 0.001	0.55 (0.24, 0.87)	0.001
P. unsure- M. normal weight	0.79 (0.31, 1.28)	0.001	0.75 (0.29, 1.21)	0.002	0.39 (0.08, 0.70)	0.013	0.44 (0.09, 0.69)	0.01	0.43 (0.15, 0.72)	0.003	0.38 (0.11, 0.66)	0.007
P. unsure- M. overweight	0.58 (−0.14, 1.31)	0.115	0.32 (−0.39, 1.02)	0.38	0.31 (−0.16, 0.77)	0.199	0.44 (−0.17, 0.74)	0.23	0.34 (−0.09, 0.76)1	0.123	0.09 (−0.33, 0.51)	0.673
Random effects												
Level 3 (School) intercept variance	0.36 (0.11, 1.10)		0.18 (0.04, 0.87)		0.25 (0.11, 0.56)		0.11 (0.03, 0.38)		0.11 (0.03,0.34)		0.04 (0.00, 0.35)	
Level 2 (Child) intercept variance	10.52 (9.30, 11.90)		8.18 (7.12, 9.41)		4.57 (4.05, 5.14)		3.72 (3.27, 4.24)		3.05 (2.66, 3.50)		2.47 (2.11, 2.89)	
Leve l1 (Occasion) intercept variance	12.75 (11.88, 13.69)		12.24 (11.39, 13.15)		5.19 (4.83, 5.57)		5.00 (4.65, 5.36)		4.65 (4.33, 4.90)		4.58 (4.26, 4.92)	

Model 1: coefficients were estimated with linear-mixed models with random intercept, with adjustment for age

Model 4: adjusted for age, ethnicity, current smoking, current alcohol consumption, special diets, weight gain anxiety, and physical activity, maternal overweight, paternal overweight, parental care, and parental control, family affluence and racism

The addition of lifestyle factors (model 3) attenuated the associations, mainly due to the addition of weight gain related anxiety. Further reductions in effect sizes were observed on adjustment for parental style and parental overweight, mainly due to maternal overweight (Full Model).

### Weight misperception and probable clinical cases

**Fig. 1:**
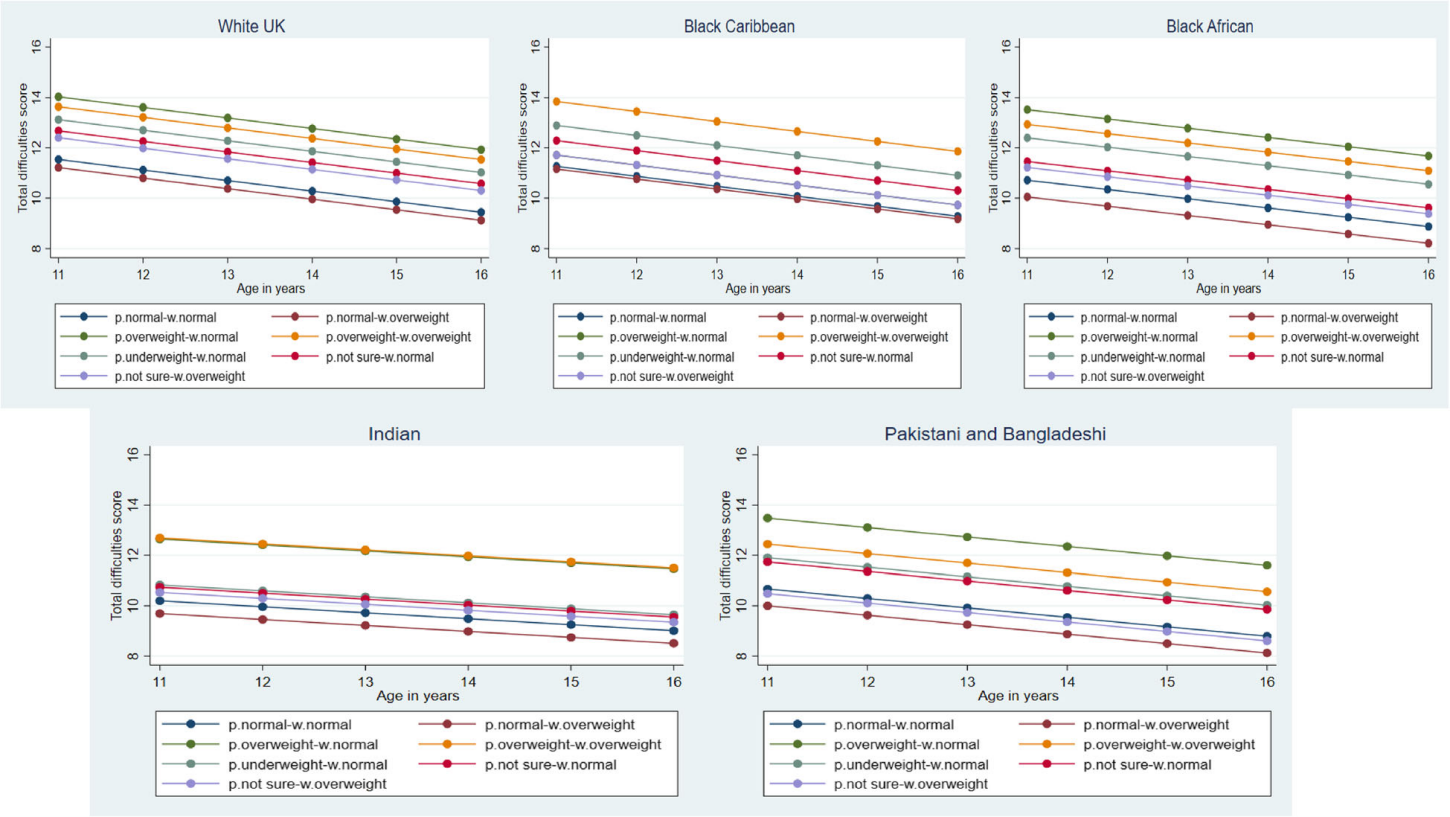
Trajectories of mean Total Difficulties Score (TDS) by weight misperception and ethnicity, adjusted for gender interaction from the age of 11–16 years. Data from the Determinants of Adolescent (now young Adult) Social well-being and Health (DASH) study. TDS means were predicted from linear-mixed models with random intercept, with adjustment of weight misperception categories, gender interaction, age, ethnicity, current smoking, current alcohol consumption, weight gain anxiety, physical activity, parental overweight, parental care, parental control, family affluence and racism

Figure 1 shows predicted mean TDS by weight misperception and ethnicity adjusted for gender interaction across adolescence, derived from the full model in Table 3. Across all ethnic groups there was a general pattern of *p.normal weight*/m.normal weight and *p.normal weight*/m.overweight being associated with the lowest mean TDS; and *p.overweight*/m.overweight and *p.overweight*/m.normal weight, associated with the highest mean TDS.

Table 4 shows gender-specific associations across adolescence between weight misperceptions and probable clinical cases measured through SDQ. Adjusted for all covariates, weight misperception categories that were independent longitudinal correlates of probable clinical cases were broadly similar to those observed for mean SDQ total, externalizing and internalizing symptoms.

**Table 4:** Females and Males: The association between weight misperception and probable clinically relevant SDQ total, externalising and internalising symptoms from 11 to 13 years to 14–16 years

	SDQ Total Difficulties (score > 17)				SDQ externalising (score > 11)				SDQ internalising (score > 9)			
	OR (95% CI)	P > |z|	OR (95% CI)	P > |z|	OR (95% CI)	P > |z|	OR (95% CI)	P > |z|	OR (95% CI)	P > |z|	OR (95% CI)	P > |z|
	Model1		Model 4		Model 1		Model 4		Model 1		Model 4	
**Females**												
*Weight perception (*vs. *P. normal weight - M. normal weight)*												
P. overweight- M. overweight	2.76 (1.66, 4.57)	< 0.001	2.01 (1.18, 3.41)	0.01	1.23 (0.62, 2.42)	0.548	1.39 (0.72, 2.72)	0.329	3.03 (1.74, 5.27)	< 0.001	1.95 (1.11, 3.43)	0.02
P. normal - M. overweight	1.00 (0.50, 2.00)	0.99	0.84 (0.41, 1.74)	0.643	0.55 (0.24, 1.26)	0.158	0.71 (0.27, 1.90)	0.499	1.14 (0.55, 2.35)	0.723	0.88 (0.42, 1.86)	0.747
P. overweight- M. normal weight	3.42 (1.88, 6.21)	< 0.001	2.06 (1.10, 3.87)	0.024	2.90 (0.97, 8.61)	0.055	1.24 (0.55, 2.78)	0.6	4.63 (2.45, 8.73)	< 0.001	2.64 (1.38, 5.05)	0.003
P. underweight- M. normal weight	2.23 (1.26, 3.96)	0.006	1.87 (1.03, 3.40)	0.039	1.86 (1.10, 3.13)	0.02	0.81 (0.35, 1.84)	0.611	2.72 (1.49, 4.96)	0.001	2.69 (1.46, 4.96)	0.002
P. unsure- M. normal weight	1.76 (1.14, 2.71)	0.011	1.39 (0.88, 2.18)	0.154	1.21 (0.71, 2.04)	0.483	0.80 (0.72, 2.27)	0.394	1.80 (1.12, 2.88)	0.015	1.34 (0.83, 2.16)	0.235
P. unsure- M. overweight	1.28 (0.68, 2.42)	0.438	1.10 (0.57, 2.11)	0.78	1.29 (0.59, 2.83)	0.518	0.81 (0.43, 2.48)	0.936	1.86 (0.97, 3.56)	0.061	1.40 (0.73, 2.69)	0.317
Random effects												
Level 3 (School) intercept	0.08 (0.01, 0.55)		0.09 (0.01, 0.63)		0.00 (0.00, 0.00)		0.00 (0.00, 0.00)		0.19 (0.05, 0.66)		0.10 (0.01, 0.69)	
Level 3 (school)> Level 2 (individual) intercept	2.74 (1.79, 4.19)		2.46 (1.55, 3.90)		3.09 (1.84, 5.18)		2.49 (1.29, 4.79)		3.20 (2.08, 4.93)		2.55 (1.58, 4.13)	
**Males**												
*Weight perception (*vs. *P. normal weight - M. normal weight)*												
P. overweight- M. overweight	3.30 (1.89, 5.74)	< 0.001	2.16 (1.25, 3.74)	0.006	1.23 (0.62, 2.42)	0.548	1.05 (0.52, 2.11)	0.89	5.63 (2.98, 10.59)	< 0.001	2.94 (1.58, 5.49)	0.001
P. normal - M. overweight	0.74 (0.37, 1.46)	0.381	0.68 (0.35, 1.30)	0.243	0.55 (0.24, 1.26)	0.158	0.60 (0.26, 1.35)	0.218	0.95 (0.43, 2.08)	0.893	0.77 (0.36, 1.68)	0.519
P. overweight- M. normal weight	1.81 (0.60, 5.46)	0.296	1.32 (0.47, 3.70)	0.597	2.90 (0.98, 8.61)	0.055	1.93 (0.66, 5.68)	0.232	4.40 (1.46, 13.29)	0.009	2.11 (0.72, 6.13)	0.17
P. underweight- M. normal weight	2.05 (1.25, 3.34)	0.004	2.34 (1.47, 3.71)	< 0.001	1.86 (1.10, 3.13)	0.02	1.82 (1.11, 3.13)	0.019	2.63 (1.46, 4.73)	0.001	2.13 (1.21, 3.75)	0.009
P. unsure- M. normal weight	1.62 (1.02, 2.56)	0.04	1.61 (1.04, 2.49)	0.033	1.21 (0.71, 2.03)	0.483	1.09 (0.64, 1.85)	0.75	1.39 (0.78, 2.46)	0.26	1.19 (0.68, 2.07)	0.549
P. unsure- M. overweight	1.51 (0.76, 2.98)	0.237	1.15 (0.60, 2.20)	0.679	1.29 (0.59, 2.83)	0.518	1.31 (0.59, 2.88)	0.507	0.99 (0.41, 2.46)	0.99	0.66 (0.27, 1.62)	0.366
Random effects												
Level 3 (School) intercept	0.08 (0.00, 0.99)		0.04 (0.00, 0.13)		0.27 (0.09, 0.80)		0.22 (0.07, 0.73)		0.00 (0.00, 0.00)		0.00 (0.00, 0.00)	
Level 3 (school)> Level 2 (individual) intercept	2.93 (1.88, 4.57)		2.63 (1.71, 4.04)		3.09 (1.84, 5.18)		2.14 (1.16, 3.95)		3.24 (1.92, 3.24)		2.00 (1.03, 3.87)	

Model 1: coefficients were estimated with linear-mixed models with random intercept, with adjustment for age

Model 4: adjusted for age, ethnicity, current smoking, current alcohol consumption, special diets, weight gain anxiety, and physical activity, maternal overweight, paternal overweight, parental care, and parental control, family affluence and racism

The addition of lifestyle factors (model 3) attenuated the associations, mainly due to the addition of weight gain anxiety in *p.overweight*/m.overweight in both genders and *p. overweight*/m.normal among females but strengthened the association in *p.underweight*/m.normal in females. Further attenuations in effect sizes were observed on adjustment for parental style (Model 4), mainly due to the addition of racism.

### Weight perception and psychological difficulty in adulthood

Table 5 shows the association between weight misperception and mean GHQ score at 21-23y, adjusted for age and gender, and additionally for ethnicity and own education level. The proportion that accurately assessed their normal weight was similar to the proportion in early adolescence (39%). The proportion that accurately assessed their overweight increased from 9% in early adolescence to 26% in early 20s. Misperceptions of weight status appeared to have decreased but small sample size prohibits reliable interpretation. Within these limitations, however, compared with those who accurately perceived their normal weight status, *p.overweight*/ m.overweight associated with a higher mean GHQ score, with little change in effect after the additional adjustments.

**Table 5:** The association between weight perception at 21–23 years and mean General Health Questionnaire score at 21–23 years

		Model 1^**a**^		Model 2^**b**^	
	N (%)	β (95% CI)	P>|z|	β (95% CI)	P >|z|
Weight perception (vs. P. normal weight - M. normal weight)					
P. normal weight - M. normal weight§	234 (39)	1		1	
P. overweight - M. overweight	154 (26)	**2.36 (1.12, 3.59)**	**< 0.001**	**2.40 (1.16, 3.62)**	**< 0.001**
P. discordant - M. normal weight	119 (20)	1.30 (−0.04, 2.64)	0.057	1.10 (−0.26, 2.45)	0.112
P. discordant - M. overweight	84 (14)	−1.17 (−2.71, 0.37)	0.135	−1.25 (−2.79, 0.29)	0.112
Age (vs. 21y)		−0.16 (− 0.78, 0.46)	0.620	− 0.15 (− 0.77, 0.47)	0.645
Female (vs. male)		**1.70 (0.70,2.69)**	**< 0.001**	**1.90 (0.89, 2.90)**	**< 0.001**
Ethnicity (vs. White UK)					
Black Caribbean				−0.70 (−2.39, 1.00)	0.420
Black African				−1.58 (−3.18, 0.03)	0.054
Indian				−1.10 (−2.86, 0.65)	0.218
Pakistani/Bangladeshi				0.67 (−0.98, 2.32)	0.424
Others				−1.58 (− 3.23, 0.07)	0.060
*Not educated to degree level (*vs. *yes)*				**1.06 (0.03, 2.09)**	**0.044**

§ Percentages do not add up to 100 due to missing values

^a^Model 1: coefficients were estimated with linear-mixed models with adjustment on age and gender

^b^Model 2: same as model 1 + adjustment for ethnicity and educational level

## Discussion

This is the first prospective UK study to evaluate the association between weight misperception and psychological symptoms in in an ethnically diverse cohort of young people. In general, weight misperception varied according to age, gender and ethnicity. At 11-13y, 46% females and 38% males did not have an accurate perception of their measured weight. The comparable figures at 14-16y were 42 and 40%. Most of these adolescents misperceived a measured normal weight. An accurate perception of normal weight was protective of against higher psychological symptoms. Misperception in adolescence among those who were measured as having a normal weight, namely a perception of underweight among males and females, unsure of weight among males, and overweight among females, was associated with higher psychological symptoms. Among those in their 20s, accurate perceptions of overweight were associated with greater psychological symptoms compared with those who accurately perceived their weight to be normal. These patterns were broadly consistent across ethnic groups, despite significant variations in weight misperceptions. Overall, these findings suggest that weight misperception was independently associated with higher levels of psychological symptoms, adjusted for gender, ethnicity, health behaviors, family environments and social adversity.

### Comparison with other studies

There are few longitudinal studies of healthy community samples of adolescents that have investigated the impact of weight misperception on psychological symptoms at a population level. These signal similar adverse impact in that perception of overweight, whether measured overweight or normal weight, was associated with a greater likelihood of a depressed mood [34]. Other studies were based on overweight young people, and though not directly comparable, signaled a protective effect of perception of normal weight status as they reported fewer depressive symptoms and higher quality of life than those who had a perception of overweight [35–37]. A national longitudinal US based study of 2738 adolescents found supportive evidence for a protective effect of a misperception of ‘average’ weight among those who had measured overweight for lower depressive symptoms 12 years later, evident in White participants only and stronger in females than in males [37].

Our findings are generally correspondent with this protective effect for those who perceived normal weight but measured overweight although the effect was not statistically significant compared with those who accurately perceived their normal weight status. Our findings on the importance of internalizing (i.e. emotional and peer-relationship problems) rather than externalizing symptoms among normal-weight females who perceived themselves to be overweight partly aligns with those from a study of Dutch adolescents aged 11–16 years [38]. Overestimation of weight relates to body dissatisfaction or greater body preoccupation [39–41] which in turn may predispose to mental health problems. The association between weight misperception and body dissatisfaction is likely to be mediated by weight-related bullying, perceived pressures to conform to socially prescribed body ideals and social comparison [42–45]. Weight misperception may be a risk factor for disordered eating in the same way that body dissatisfaction has been found to be [46–48].

### Strengths and limitations

The DASH study is the largest longitudinal study of ethnically diverse young people in the UK designed to examine ethnic inequalities in health. Self-ascribed ethnicity was compared with ethnicity of parents and grandparents to check for inconsistencies. The sample is well characterized in relation to diversity and psychosocial measures, including parent-child relationships and multidimensional measures of socioeconomic disadvantage. Participant and item response rates were also very high, aided by enormous community support and regular updated training of research assistants during the data collection period. A small percentage of high SDQ scorers (1–2%), were lost to follow-up in adolescence but this is unlikely to explain these findings. The lack of assessment of eating-disordered behavior is another limitation, given the strong associations between eating-disordered behavior and body dissatisfaction, and between eating disordered behavior and higher psychological symptoms [49, 50]. A further limitation of this study is the lack of information on weight-related bullying provided that it is related to weight misperception and increased psychological symptoms [44, 51, 52]. The sample size of the pilot study at 21-23y was small for robust analysis of the weight misperception categories but gave some indication of potential patterns in need of further enquiry in other studies. Confounding bias could be expected from unmeasured factors (e.g. weight stigma).

### Implications

These findings support the need for system level interventions (e.g. school, community, policy system interventions) to promote healthy body image. Currently, school-based health interventions tend to focus on ‘problems’ and ‘risks’ associated with overweight/obesity and on the behaviours (e.g, those relating to diet and physical activity) required to maintain a ‘healthy’ body weight. For example, national school programs track anthropometry and school food environments focus on reducing sugar sweetened beverages, salt and fat intake. Integrated school prevention programs with community components (e.g. community clubs, primary care) have the potential to engage young people and the providers of care (e.g. parents, teachers, practitioners) in programmes which promote not only body weight and physical health but also weight misperception and/or body dissatisfaction and mental health [53]. Others have made cogent arguments for interventions to engage with issues such as the internalisation of thin and muscular body ideas, and social comparisons on body image development [54, 55], and for the need to understand the underlying mental representation of human bodies [56]. Our findings support engagement with other determinants of weight misperception such as weight gain anxiety, parenting and maternal weight. Given the contextual complexities of young peoples’ lives (e.g. intersectional influences of ethnicity, gender, socio-economic circumstances), formative research is needed to obtain adolescents’ perspectives on how concepts such as misperceptions and body dissatisfaction can be meaningfully translated into prevention programmes that will shape their future health and well-being.

## Conclusions

This study highlights that weight misperception is an important determinant of increased psychological symptoms and probable clinical cases in young people, regardless of ethnicity, with an accurate perception of normal weight status being protective. Culturally targeted interventions should be considered to promote healthy perceptions of body image.

## Supplementary information

**Additional file 1 Supplementary Table 1a.** Females - Sample characteristics from 11 to 13 years to 14–16 years, N (%). The Determinants of Adolescent Social well-being and Health study. **Supplementary Table 1b.** Males - Sample characteristics from 11 to 13 years to 14–16 years, N (%). The Determinants of Adolescent Social well-being and Health study. **Supplementary Table 2:** Females and Males: The association between weight misperception and mean SDQ total, externalising and internalising scores from 11 to 13 years to 14–16 years (Models 2 and 3). **Supplementary Table 3:** Females and Males: The association between weight misperception and probable clinically relevant SDQ total, externalising and internalising symptoms from 11 to 13 years to 14–16 years (Models 2 and 3).

## Data Availability

The datasets are not publicly available due to ethical considerations. The authors will support access to the data where requests are reasonable and consistent with the ethical approval of the Multicentre Research Ethics Committee.

## Abbreviations

DASH: Determinants of young Adults Social well-being and Health study
BMI: Body Mass Index
FAS: Family Affluence Scale
GHQ: General Health Questionnaire
OR: Odds Ratio
PBI: Parental Bonding Instrument
SDQ: Strengths and Difficulties Questionnaire
SEC: Socioeconomic Circumstances
TDS: Total Difficulties Score
UK: United Kingdom; US: United States
