# Supplementary material for: Weight misperception and psychological symptoms from adolescence to young adulthood: longitudinal study of an ethnically diverse UK cohort
Source: BMC Public Health. 2020 May 18;20:712. doi: 10.1186/s12889-020-08823-1 (PMC7236343; doi:10.1186/s12889-020-08823-1)
Supplement: Supplementary file 1 — Additional file 1 Supplementary Table 1a. Females - Sample characteristics from 11 to 13 years to 14–16 years, N (%). The Determinants of Adolescent Social well-being and Health study. Supplementary Table 1b. Males - Sample characteristics from 11 to 13 years to 14–16 years, N (%). The Determinants of Adolescent Social well-being and Health study. Supplementary Table 2: Females and Males: The association between weight misperception and mean SDQ total, externalising and internalising scores from 11 to 13 years to 14–16 years (Models 2 and 3). Supplementary Table 3: Females and Males: The association between weight misperception and probable clinically relevant SDQ total, externalising and internalising symptoms from 11 to 13 years to 14–16 years (Models 2 and 3). [file 12889_2020_8823_MOESM1_ESM.docx]

| **Supplementary Table 1a.** Females - Sample characteristics from 11-13 years to 14-16 years, N (%). The Determinants of Adolescent Social well-being and Health study. | | | | | | | | | | | | | | | | | | | | |
| --- | --- | --- | --- | --- | --- | --- | --- | --- | --- | --- | --- | --- | --- | --- | --- | --- | --- | --- | --- | --- |
|  | All  (N=1,493) | | | | White British  (N=383) | | | | Black Caribbean  (N = 351) | | | Black African  (N = 446) | | | Indian  (N = 172) | | Pakistani/Bangladeshi  (N = 141) | | | |
|  | 11-13 yrs | | 14-16 yrs | 11-13 yrs | | | | 14-16yrs | 11-13 yrs | | 14-16 yrs | 11-13 yrs | | 14-16 yrs | 11-13 yrs | 14-16yrs | 11-13 yrs | | | 14-16 yrs |
| Total Difficulties Score (mean ± SD) | 11.0± 5.2 | 10.9 ± 4.9 | | 11.1 ±5.1 | | | 11.4±4.9 | | 11.4±5.2 | | 11.0± 4.9 | 11.1±5.3 | | 10.9±4.9 | 9.4 ±4.6* | 9.7±4.5* | 11.3±5.4 | | | 11.0±5.0 |
| TDS >17 | 210 (14) | 198 (13) | | 54 (14) | | | 59(15) | | 56 (16) | | 47 (13) | 61 (14) | | 58 (13) | 14 (8) | 13 (8) | 25 (18) | | | 21 (15) |
| P. normal weight - M. normal weight | 508 (34) | 591 (40) | | 160 (42) | | | 165 (43) | | 112 (32)^ | | 141 (40) | 138 (31)^ | | 173 (39) | 55 (32) | 66 (38) | 43 (31) | | | 46 (33) |
| P. overweight- M. overweight | 152 (10) | 176 (12) | | 33 (9) | | | 32 (8) | | 46 (13) | | 52 (15) | 50 (11) | | 65 (15)^ | 16 (9) | 15 (9) | 7 (5) | | | 12 (9) |
| P. normal weight - M. overweight | 139 (9) | 66 (4) | | 26 (7) | | | 9 (2)* | | 50 (14)^ | | 25 (7)^ | 46 (10)^ | | 23 (5) | 11 (6) | 5 (3) | 6 (4) | | | 4 (3) |
| P. overweight - M. normal | 68 (5) | 98 (7) | | 24 (6) | | | 34 (9) | | 7 (2)^ | | 17 (5) | 17 (4) | | 21 (5) | 9 (5) | 12 (7) | 11 (8) | | | 14 (10) |
| P. underweight - M. normal weight | 117 (8) | 111 (7) | | 32 (8) | | | 22 (6) | | 26 (7) | | 23 (7) | 31 (7) | | 31 (7) | 12 (7) | 18 (10) | 16 (11) | | | 17 (12) |
| P. unsure- M. normal weight | 246 (16) | 260 (17) | | 65 (17) | | | 66 (17) | | 38 (11) | | 50 (14) | 64 (14) | | 71 (16)^ | 43 (25) | 38 (22) | 36 (26) | | | 35 (25) |
| P. unsure- M. overweight | 115 (8) | 101 (7) | | 19 (5) | | | 16 (4) | | 36 (10) | | 24 (7) | 33 (7) | | 44 (10) | 15 (9) | 11 (6) | 12 (9) | | | 6 (4) |
| Not stated | 158 (10) | 90 (6) | | 24 (6) | | | 39 (10) | | 36 (10) | | 19 (5) | 67 (15) | | 18 (4) | 11 (6) | 7 (4) | 10 (7) | | | 7 (5) |
| Normal weight | 1,042 (70) | 1,064 (71) | | 300 (78) | | | 287 (75) | | 208(60)^ | | 232(66) | 290 (65)^ | | 297 (67) | 129 (75) | 136 (79) | 115 (82) | | | 112 (79) |
| Overweight | 451 (30) | 344 (23)* | | 83 (22) | | | 57 (15) | | 143 (41)^ | | 101 (29)^ | 156 (35)^ | | 133 (30)^ | 43 (25) | 31 (18) | 26 (18) | | | 22 (16) |
| Not stated | 0 (0) | 85 (6) | | (0) | | | 39 (10) | | (0) | | 18 (5) | (0) | | 16 (4) | (0) | 5 (3) | (0) | | | 7 (5) |
| Weight gain anxiety (no) | 611 (41) | 634 (42) | | 171 (45) | | | 143 (37) | | 162 (46) | | 179 (51)^ | 165 (37) | | 197 (44) | 67 (39) | 67 (39) | 46 (33) | | | 48 (34) |
| Weight gain anxiety (yes) | 737 (49) | 853 (57) | | 192 (50) | | | 238 (62) | | 156 (44) | | 170 (48)^ | 211 (47) | | 248 (56) | 93 (54) | 104 (60) | 85 (60) | | | 93 (66) |
| Not stated | 145 (10) | 6 (0) | | 20 (5) | | | 2 (1) | | 33 (9) | | 2 (1) | 70 (16) | | 1 (0) | 12 (7) | 1 (1) | 10 (7) | | | 0 (0) |
| Special diet (no) | 831 (56) | 932 (62) | | 289 (75) | | | 312 (81) | | 246 (70) | | 282 (80) | 255 (57)^ | | 293 (66)^ | 38 (22)^ | 40 (23)^ | 3 (2)^ | | | 5 (4)^ |
| Special diet (yes) | 522 (35) | 556 (37) | | 74(19) | | | 69 (18) | | 72 (21) | | 67 (19) | 123 (28)^ | | 152 (34)^ | 124 (72)^ | 132(77)^ | 129(91)^ | | | 136 (96)^ |
| Not stated | 140 (9) | 5 (0) | | 20 (5) | | | 2 (1) | | 33 (9) | | 2 (1) | 68 (15) | | 1 (0) | 10 (6) | 0 (0) | 9 (6) | | | 0 (0) |
| Smoking (no) | 1,242 (83) | 1,296 (87) | | 341 (89) | | 272 (71) | | | 292 (83) | 302 (86)^ | | 352 (79)^ | 421 (94)^ | | 140 (81) | 165(96)^ | 117 (83) | | 136 (97)^ | |
| Smoking (yes) | 33 (2) | 187 (13) | | 15 (4) | | 108 (28)* | | | 13 (4) | 46 (13)^* | | 3 (1)^ | 22 (5)^* | | 1(1) | 7 (4)^ | 1 (1) | | 4 (3)^ | |
| Not stated | 218 (15) | 10 (1) | | 27 (7) | | 3 (1) | | | 46 (13) | 3 (1) | | 91 (20) | 3 (1) | | 31 (18) | 0 (0) | 23 (16) | | 1 (1) | |
| Alcohol consumption (no) | 863 (58) | 665 (44) | | 184 (48) | | 60 (16) | | | 150 (43) | 104 (30)^ | | 267 (60)^ | 264 (59)^ | | 140 (81)^ | 108(63)^ | 122(87)^ | | 129 (91)^ | |
| Alcohol consumption (yes) | 431 (29) | 812 (54) | | 176 (46) | | 320 (84)* | | | 150 (43) | 243 (69)^* | | 88 (20)^ | 175 (39)^* | | 16 (9)^ | 64 (37)^* | 1(1)^ | | 10 (7)^ | |
| Not stated | 199 (13) | 16 (1) | | 23 (6) | | 3 (1) | | | 51 (15) | 4 (1) | | 91 (20) | 7 (2) | | 16 (9) | 0 (0) | 18 (13) | | 2 (1) | |
| *Physical activity (per week)* |  |  | |  | |  | | |  |  | |  |  | |  |  |  | |  | |
| 5 times | 509 (34) | 167 (11) | | 135 (35) | | 35 (9) | | | 131 (37) | 42 (12) | | 141 (32) | 47 (11) | | 52 (30) | 20 (12) | 50 (35) | | 23 (16) | |
| 3 or 4 times | 383 (26) | 437 (29) | | 102 (27) | | 118 (31) | | | 85 (24) | 97 (28) | | 125 (28) | 130 (29) | | 36 (21) | 55 (32) | 35 (25) | | 37 (26) | |
| 2 times | 225 (15) | 343 (23) | | 69(18) | | 90 (24) | | | 53 (15) | 78 (22) | | 57 (13) | 99 (22) | | 29 (17) | 41 (24) | 17 (12) | | 35 (25) | |
| 1 time | 174 (12) | 355 (24) | | 43 (11) | | 101 (26) | | | 47 (13) | 85 (24) | | 48 (11) | 106 (24) | | 23 (13) | 39 (23) | 13 (9) | | 24 (17) | |
| None | 101 (7) | 183 (12) | | 18 (5) | | 36 (9) | | | 14 (4) | 46 (13) | | 31 (7) | 63 (14) | | 22 (13)^ | 17 (1) | 16 (11) | | 21 (15) | |
| Not stated | 101 (7) | 8 (1) | | 16 (4) | | 3 (1) | | | 21 (6) | 3 (1) | | 44 (10) | 1 (0) | | 10 (6) | 0 (0) | 10 (7) | | 1 (1) | |
| Maternal overweight (no) | 1,080 (72) | 1,173 (79) | | 292 (76) | | 308 (80) | | | 248 (71) | 283 (81) | | 324 (73) | 350 (78) | | 124 (72) | 130 (76) | 92 (65) | | 102 (72) | |
| Maternal overweight (yes) | 123 (8) | 194 (13) | | 26 (7) | | 46 (12) | | | 31 (9) | 40 (11) | | 33 (7) | 58 (13) | | 16 (9) | 22 (13) | 17 (12) | | 28 (20) | |
| Not stated | 290 (19) | 126 (8) | | 65 (17) | | 29 (8) | | | 72 (21) | 28 (8) | | 89 (20) | 38 (9) | | 32 (19) | 20 (12) | 32 (23) | | 11 (8) | |
| Paternal overweight (no) | 838 (56) | 1,087 (73) | | 224 (58) | | 279 (73) | | | 140(40)^ | 241 (69) | | 251 (56) | 329 (74) | | 131 (76)^ | 131 (76) | 92 (65) | | 107 (76) | |
| Paternal overweight (yes) | 65 (4) | 129 (9) | | 25 (7) | | 42 (11) | | | 5 (1)^ | 22 (6)* | | 12 (3) | 30 (7) | | 11 (6) | 18 (10) | 12 (9) | | 17 (12) | |
| Not stated | 590 (40) | 277 (19) | | 134 (35) | | 62 (16) | | | 206 (59) | 88 (25) | | 183 (41) | 87 (20) | | 30 (17) | 23 (13) | 37 (26) | | 17 (12) | |
| *Parental care* |  |  | |  | |  | | |  |  | |  |  | |  |  |  | |  | |
| High care tertile | 575 (39) | 301 (20) | | 168 (44) | | 93 (24)* | | | 137 (39) | 55 (16)^* | | 149 (33)^ | 73 (16)^* | | 67 (39) | 43 (25) | 54 (38) | | 37 (26) | |
| Medium care tertile | 417 (28) | 392 (26) | | 120 (31) | | 111 (29) | | | 85 (24) | 97 (28) | | 121 (27) | 106 (24) | | 52 (30) | 48 (28) | 39 (28) | | 30 (21) | |
| Low care tertile | 430 (29) | 784 (53) | | 83 (22) | | 175 (46)* | | | 105 (30) | 196 (56)* | | 149 (33)^ | 260 (58)^* | | 49 (28) | 80(47)* | 44 (31) | | 73 (52)* | |
| Not stated | 71 (5) | 4 (1) | | 12 (3) | | 3 (1) | | | 24 (7) | 3 (1) | | 27 (6) | 7 (2) | | 4 (3) | 1 (1) | 4 (3) | | 1 (1) | |
| *Parental control* |  |  | |  | |  | | |  |  | |  |  | |  |  |  | |  | |
| Low control tertile | 398 (27) | 427(29) | | 163 (43) | | 166 (43) | | | 80 (23)^ | 91 (26)^ | | 86 (19)^ | 102 (23)^ | | 38 (22)^ | 38 (22)^ | 31 (22)^ | | 30 (21)^ | |
| Medium control tertile | 536 (36) | 449 (30) | | 142 (37) | | 113 (30) | | | 125 (36) | 109 (31) | | 152 (34) | 128 (29) | | 70 (41) | 56 (33) | 47 (33)^ | | 43 (31) | |
| High control tertile | 491 (33) | 601 (40) | | 68 (18) | | 99 (26)* | | | 126(36)^ | 148 (42)^* | | 178 (40)^ | 208 (47) | | 59 (34)^ | 78 (45) | 60 (43)^ | | 68 (48) | |
| Not stated | 68 (5) | 16 (1) | | 10 (3) | | 5 (1) | | | 20 (6) | 3 (1) | | 30 (7) | 8 (2) | | 5 (3) | 0 (0) | 3 (2) | | 0 (0) | |
| Parental employment |  |  | |  | |  | | |  |  | |  |  | |  |  |  | |  | |
| ≥ 1 employed | 1,131 (76) | 1,187 (80) | | 299 (78) | | 310 (81) | | | 264 (75) | 297 (85)* | | 328 (74) | 345 (77) | | 152 (88)^ | 149 (87) | 88 (62)^ | | 86 (61)^ | |
| Neither employed | 273 (18) | 204 (14) | | 65 (17) | | 35 (9)* | | | 56 (16) | 30 (9)* | | 86 (19) | 80 (18)^ | | 14 (8)^ | 14 (8) | 52 (37)^ | | 45 (32)^ | |
| Other | 41 (3) | 1 (1) | | 7 (2) | | 0 (0) | | | 18 (5) | 0 (0) | | 14 (3) | 1 (0) | | 2 (1) | 0 (0) | 0 (0) | | 0 (0) | |
| Not stated | 48 (3) | 101 (7) | | 12 (3) | | 38 (10) | | | 13 (4) | 24 (7) | | 18 (4) | 20 (4) | | 4 (2) | 9 (5) | 1 (1) | | 10 (7) | |
| Family Affluence |  |  | |  | |  | | |  |  | |  |  | |  |  |  | |  | |
| High | 773 (52) | 959 (64) | | 243 (63) | | 272 (71) | | | 153(44)^ | 202 (58)^* | | 232 (52)^ | 281 (63)* | | 90 (52) | 123(72)* | 55 (39)^ | | 81 (57)^* | |
| Medium | 466 (31) | 471 (32) | | 93 (24) | | 92 (24) | | | 123(35)^ | 130 (37)^ | | 136 (30) | 149 (33)^ | | 53 (31) | 44 (26)^ | 61 (43)^ | | 56(40)^ | |
| Low | 57 (4) | 21 (1) | | 16 (4) | | 6 (2) | | | 21 (6) | 12 (3) | | 12 (3) | 3 (1) | | 3 (2) | 0 (0) | 5 (4) | | 0 (0) | |
| Not stated | 197 (13) | 42 (3) | | 31 (8) | | 13 (3) | | | 54 (15) | 7 (2) | | 66 (15) | 13 (3) | | 26 (15) | 5 (3) | 20 (14) | | 4 (3) | |
| Reported racism (no) | 964 (65) | 959 (64) | | 303 (79) | | 272 (71) | | | 219(62)^ | 202 (58)^ | | 252 (57)^ | 281 (63)^ | | 112 (65)^ | 123(72)^ | 78 (55)^ | | 81 (58)^ | |
| Reported racism(yes) | 271 (18) | 471 (32) | | 47 (12) | | 92 (24) | | | 69 (20) | 130 (37)^* | | 82 (18) | 149 (33)^* | | 35 (20) | 44 (26)^ | 38 (27)^ | | 56 (40)^ | |
| Not stated | 258 (17) | 21 (1) | | 33 (9) | | 6 (2) | | | 63 (18) | 12 (3) | | 112 (25) | 3 (1) | | 25 (15) | 0 (0) | 25 (18) | | 0 (0) | |
| **P* < 0.05compared with 11-13 years; ^*P* < 0.05 compared with White British. | | | | | | | | | | | |  | |  |  |  |  |  | | |

| **Supplementary Table 1b.** Males - Sample characteristics from 11-13 to14-16 years percentage, N (%). The Determinants of Adolescent Social well-being and Health study | | | | | | | | | | | | | | | | | | | | | |
| --- | --- | --- | --- | --- | --- | --- | --- | --- | --- | --- | --- | --- | --- | --- | --- | --- | --- | --- | --- | --- | --- |
|  | All  (N=1,734) | | | | White British  (N=484) | | | | Black Caribbean  (N = 344) | | | Black African  (N = 372) | | | | | Indian  (N = 224) | | Pakistani/Bangladeshi  (N = 310) | | |
|  | 11-13 yrs | | 14-16 yrs | 11-13 yrs | | | | 14-16 yrs | 11-13 yrs | | 14-16 yrs | | 11-13 yrs | | | 14-16 yrs | 11-13 yrs | 14-16yrs | 11-13 yrs | | 14-16 yrs |
| Total Difficulties Score (mean ± SD) | 10.5± 5.1 | 9.7 ± 4.7* | | 11.3 ± 5.0 | | | 10.3*±4.6 | | 10.8± 5.1 | | 9.6*^± 4.6 | 10.0^ ±4.6 | | | 8.95*^± 4.5 | | 10.1^ ± 5.8 | 9.7^± 5.2 | 10.2^ ±5.1 | | 9.5*^±4.5 |
|  |  |  | |  | | |  | |  | |  |  | | |  | |  |  |  | |  |
| P. normal weight - M. normal weight | 711 (41) | 820 (47) | | 215 (44) | | | 239 (50) | | 149 (43) | | 165 (48) | 143 (38) | | | 179 (48)^* | | 77 (34) | 92 (41) | 127 (41) | | 145 (47) |
| P. overweight- M. overweight | 130 (8) | 129 (7) | | 45 (9) | | | 44 (10) | | 20 (6) | | 26 (8) | 17 (5) | | | 16 (4)^ | | 22 (10) | 17 (8) | 26 (8) | | 26 (8) |
| P. normal weight - M. overweight | 142 (8) | 117 (7) | | 34 (7) | | | 19 (4) | | 33 (10) | | 31 (9)^ | 40 (11) | | | 30 (8) | | 16 (7) | 11 (5) | 19 (6) | | 26 (8) |
| P. overweight - M. normal | 35 (2) | 28 (2) | | 11 (2) | | | 11 (3) | | 4 (1) | | 4 (1) | 6 (2) | | | 5(1) | | 8 (4) | 3 (1) | 6 (2) | | 5 (2) |
| P. underweight - M. normal weight | 151 (9) | 210 (12) | | 38 (8) | | | 48 (10) | | 18 (5) | | 35 (10) | 34 (9) | | | 53 (14) | | 31 (14) | 37(17) | 30 (10) | | 37 (12) |
| P. unsure weight - M. normal weight | 225 (13) | 246 (14) | | 61 (13) | | | 54 (11) | | 31 (9) | | 51 (15) | 51 (14) | | | 52 (14) | | 37 (17) | 40(18) | 45 (15) | | 49 (16) |
| P. unsure weight - M. overweight | 111 (6) | 81 (5) | | 33 (7) | | | 21 (4) | | 26 (8) | | 13 (4) | 17 (5) | | | 19 (5) | | 13 (6) | 15 (7) | 22 (7) | | 13 (4) |
| Not stated | 228 (13) | 101 (6) | | 47 (10) | | | 48 (10) | | 63 (18) | | 19 (6) | 64 (17) | | | 16 (4) | | 19 (9) | 9 (4) | 35 (11) | | 9 (3) |
| Normal weight | 1,287(74) | 1,310 (76) | | 361 (75) | | | 353 (73) | | 250 (73) | | 256 (74) | 283(76) | | | 291 (78) | | 164(73) | 173 (77) | 229 (18) | | 237(76) |
| Overweight | 447(26) | 332 (19)* | | 123 (25) | | | 84 (17)* | | 94(27) | | 71 (21) | 89(24) | | | 67 (18) | | 60(27) | 44 (20) | 81 (26) | | 66 (21) |
| Not stated | 1 (0) | 92 (5) | |  | | | 47 (10) | |  | | 17 (5) |  | | | 14 (4) | |  | 7 (3) |  | | 7 (2) |
| Weight gain anxiety (no) | 937(54) | 1,265 (73) | | 281(58) | | | 356 (74) | | 191(56) | | 252 (73) | 210(56) | | | 294 (79) | | 114(51) | 147 (66) | 141(45) | | 216 (70) |
| Weight gain anxiety (yes) | 585 (34) | 459 (26) | | 160 (33) | | | 127 (26) | | 87 (25) | | 89 (26) | 108 (29) | | | 77 (21) | | 92(41) | 75 (33) | 138 (45) | | 91 (29) |
| Not stated | 43(9) | 10 (1) | | 43(9) | | | 1 (0) | | 66(19) | | 3(1) | 54 (15) | | | 1(0) | | 18 (8) | 2(1) | 31 (10) | | 3 (1) |
| Special diet (no) | 842 (48) | 1,041 (60) | | 375(77) | | | 444 (92) | | 223(65) | | 291 (85) | 194(52) | | | 242 (65) | | 37(17) | 44 (20) | 13(4) | | 20 (6) |
| Special diet (yes) | 685 (40) | 685 (40) | | 67(14) | | | 39 (8) | | 54(16) | | 50 (15) | 125(34) | | | 129 (35) | | 170(76) | 179 (80) | 269(87) | | 288 (92) |
| Not stated | 207 (12) | 8 (0) | | 42 (9) | | | 1 (0) | | 67(19) | | 3 (1) | 53(14) | | | 1 (0) | | 17(8) | 1(0) | 28(9) | | 2 (1) |
| Smoking (no) | 1,362 (79) | 1,555 (90) | | 413 (85) | | 400 (83) | | | 248(72) | 316 (92) | | 277(75) | | 354(95) | | | 172(77) | 210 (94) | 252(81) | 274 (88) | |
| Smoking (yes) | 19 (1) | 164 (9)* | | 9 (1) | | 81 (17)^* | | | 5(1) | 25 (7^)* | | 1(0) | | 12 (3)^ | | | 0(0) | 13(6)^* | 4(1) | 33 (11)^ | |
| Not stated | 353 (20) | 16 (1) | | 62 (13) | | 3 (1) | | | 91 (26) | 3(1) | | 94 (25) | | 6 (2) | | | 52(23) | 1(0) | 54 (17) | 3 (1) | |
| Alcohol consumption (no) | 979 (56) | 956 (55) | | 175 (36) | | 121 (25) | | | 142 (41) | 134 (39) | | 231 (62) | | 251 (67) | | | 171(76) | 154 (69) | 260 (84) | 296 (95) | |
| Alcohol consumption (yes) | 436 (25) | 761 (43)* | | 248(51) | | 361 (75)^* | | | 114(33)^ | 205 (56)^* | | 51 (14)^ | | 115 (31)^* | | | 16 (7)^ | 68 (31)^* | 7 (2)^ | 12 (4)^ | |
| Not stated | 319 (18) | 17 (1) | | 61(13) | | 2 (0) | | | 88(26) | 5 (1) | | 90(24) | | 6 (2) | | | 37(17) | 2 (1) | 43(14) | 2 (1) | |
| *Physical activity (per week)* |  |  | |  | |  | | |  |  | |  | |  | | |  |  |  |  | |
| 5 times | 868(50) | 297 (17) | | 223 (46) | | 68 (14) | | | 175(51) | 56 (16) | | 191(51) | | 66 (18) | | | 117(52) | 42 (19) | 162(52) | 65 (21) | |
| 3 or 4 times | 435(25) | 672 (39) | | 145(30) | | 194 (40) | | | 77(22) | 133 (39) | | 85 (23) | | 147 (40) | | | 53(24) | 94 (42) | 75(24) | 104 (34) | |
| 2 times | 146(25) | 415 (24) | | 47 (10) | | 126 (26) | | | 23 (7) | 81 (24) | | 34(9) | | 84 (23) | | | 18(8) | 50 (22) | 24(8) | 74 (24) | |
| 1 time | 76 (4) | 266 (15) | | 31 (6) | | 73 (15) | | | 13(4) | 57 (17) | | 10 (3) | | 54 (15) | | | 8(4) | 31 (14) | 14(5) | 51 (16) | |
| None | 28(2) | 71 (4) | | 10(2) | | 21 (4) | | | 5(1) | 13 (4) | | 5 (1) | | 18 (5) | | | 4 (2) | 6 (3) | 4 (1) | 13 (4) | |
| Not stated | 181 (10) | 13 (1) | | 28 (6) | | 2 (0) | | | 51 (15) | 4 (1) | | 47(13) | | 3 (1) | | | 24 (11) | 1 (0) | 31(10) | 3 (1) | |
| Maternal overweight (no) | 1,369 (79) | 1,413 (81) | | 393 (81) | | 396 (81) | | | 272 (79) | 288 (84) | | 289 (75) | | 307 (83) | | | 186 (83) | 180 (80) | 238 (77) | 242 (78) | |
| Maternal overweight (yes) | 110 (6) | 133 (8) | | 26(5) | | 38 (8) | | | 17(5) | 21 (6) | | 28 (8) | | 23 (6) | | | 12(5) | 23 (10) | 27(9) | 28 (9) | |
| Not stated | 255 (15) | 188 (11) | | 65 (13) | | 50 (10) | | | 55 (16) | 35 (10) | | 64(17) | | 42 (11) | | | 26(12) | 21 (9) | 45(15) | 40 (13) | |
| Paternal overweight (no) | 1,113 (64) | 1,319 (76) | | 318 (66) | | 375 (77) | | | 184(53) | 268 (78) | | 210 (56) | | 263 (71) | | | 177(79) | 176(79) | 224 (72) | 237 (76) | |
| Paternal overweight (yes) | 76 (4) | 116 (7) | | 27(6) | | 46 (10) | | | 6(2)^ | 11 (3)^ | | 9 (2) | | 13 (3)^ | | | 15 (7) | 20(9) | 19 (6) | 26 (8) | |
| Not stated | 545(31) | 299 (17) | | 139 (29) | | 63 (13) | | | 154 (45) | 65 (19) | | 153 (41) | | 96 (26) | | | 32(14) | 28 (13) | 67(22) | 47 (15) | |
| *Parental care* |  |  | |  | |  | | |  |  | |  | |  | | |  |  |  |  | |
| High care tertile | 657(38) | 445 (26) | | 181(37) | | 124 (26)* | | | 129 (38) | 81 (24)* | | 134 (36) | | 85 (23)* | | | 92 (41) | 67(30) | 121(39) | 88 (28) | |
| Medium care tertile | 479(28) | 492 (28) | | 163 (34) | | 141 (29) | | | 80 (23)* | 92 (27) | | 91 (24)* | | 101 (27) | | | 54(24) | 71(32) | 91 (29) | 87 (28) | |
| Low care tertile | 501(29) | 775 (45) | | 125(26) | | 217 (45)* | | | 105 (31) | 165 (48)* | | 117 (31) | | 177 (48)* | | | 66(29) | 85(38) | 88 (28) | 131 (42)* | |
| Not stated | 97 (6) | 22 (1) | | 15 (3) | | 2 (0) | | | 30 (9) | 6 (2) | | 20 (8) | | 9 (2) | | | 12 (5) | 1 (0) | 10 (3) | 4 (1) | |
| *Parental control* |  |  | |  | |  | | |  |  | |  | |  | | |  |  |  |  | |
| Low control tertile | 459 (26) | 556 (32) | | 169 (35) | | 217(45)* | | | 109 (32) | 114 (33)* | | 75 (20)^ | | 91 (24)* | | | 51 (23)^ | 65 (29)* | 55 (18)^ | 69 (22)* | |
| Medium control tertile | 629 (36) | 625 (36) | | 193 (40) | | 170 (35) | | | 109 (32) | 117 (34) | | 130 (35) | | 138 (37) | | | 75 (33) | 78 (35) | 122 (39) | 122 (39) | |
| High control tertile | 547 (32) | 527(30) | | 104 (21) | | 95 (20) | | | 97 (28) | 106 (31)* | | 137 (37)* | | 132 (35)* | | | 86 (34)* | 79 (35)* | 123 (40)* | 115 (37)* | |
| Not stated | 99 (6) | 26 (2) | | 18 (4) | | 2 (0) | | | 29 (8) | 7 (2) | | 30 (8) | | 11 (3) | | | 12 (5) | 2 (1) | 10 (3) | 4 (1) | |
| *Parental employment* |  |  | |  | |  | | |  |  | |  | |  | | |  |  |  |  | |
| ≥ 1 employed | 1,333 (77) | 1,398 (81) | | 408 (84) | | 397 (82) | | | 276 (80) | 293 (82) | | 261 (70)* | | 285 (77) | | | 180 (80) | 196 (88) | 208 (67)* | 227 (73)* | |
| Neither employed | 302 (17) | 245 (14) | | 60 (12) | | 38 (8) | | | 50 (15) | 38 (8) | | 69 (19)* | | 71 (19)* | | | 35 (16) | 23 (10) | 88 (28)* | 76 (25)* | |
| Other | 31 (2) | 91 (5) | | 4 (1) | | 49 (10) | | | 9 (3) | 49 (10) | | 15 (4) | | 16 (4) | | | 1 (0) | 5 (2) | 2 (1) | 7 (2) | |
| Not stated | 68 (4) | 0 (0) | | 12 (2) | | 0 (0) | | | 9 (3) | 0 (0) | | 27 (7) | | 0 () | | | 8 (4) | 0 (0) | 12 (4) | (0) | |
| *Family Affluence* |  |  | |  | |  | | |  |  | |  | |  | | |  |  |  |  | |
| High | 970 (56) | 1,192 (69) | | 306 (63) | | 341 (70) | | | 169(49)* | 210 (61)^* | | 196 (53)* | | 252 (68)^ | | | 124 (55) | 161(72)^ | 175 (56) | 228 (74)^ | |
| Medium | 441 (25) | 452 (26) | | 114 (24) | | 123 (25) | | | 98 (28) | 106 (31) | | 95 (26) | | 99 (27) | | | 52 (23) | 52 (23) | 82 (26) | 72 (23) | |
| Low | 62 (4) | 33(2) | | 21 (4) | | 10 (2) | | | 15 (4) | 12 (3) | | 11 (3) | | 5 (1) | | | 5 (2) | 3 (1) | 10 (3) | 3 (1) | |
| Not stated | 261 (15) | 57 (3) | | 43 (9) | | 10 (2) | | | 62 (18) | 16 (5) | | 70 (19) | | 16 (5) | | | 43 (19) | 7 (2) | 43 (14) | 7 (2) | |
| Reported racism (no) | 1,004 (58) | 1,234 (71) | | 339 (70) | | 386 (80) | | | 188 (55) | 234 (68) | | 180 (48) | | 241 (65) | | | 126 (56) | 151 (67) | 171 (55) | 222 (72) | |
| Reported racism(yes) | 323 (19) | 482 (28) | | 66 (14) | | 96 (20) | | | 51 (15) | 104 (30)^* | | 77 (21) | | 126 (34)^* | | | 42 (19) | 71 (32)^* | 87 (28)^ | 85 (27) | |
| Not stated | 407 (23) | 18 (1) | | 79 (16) | | 2 (0) | | | 105 (31) | 6 (2) | | 115 (31) | | 5 (1) | | | 56 (25) | 2 (1) | 52 (17) | 3 (1) | |
| **P* < 0.05compared with 11-13 years; ^*P* < 0.05 compared with White British. | | | | | | | | | | | | |  | | |  |  |  |  |  | |

**Supplementary Table 2:** Females and Males: The association between weight misperception and mean SDQ total, externalising and internalising scores from 11-13 years to 14-16 years (Models 2 and 3)

|  | **Females** | | | | | | | | | | | |
| --- | --- | --- | --- | --- | --- | --- | --- | --- | --- | --- | --- | --- |
|  | **SDQ total difficulties score** | | | | **SDQ externalising difficulties score** | | | | **SDQ internalising difficulties score** | | | |
|  | B (95% CI) | **P > \|z\|** | B (95% CI) | **P > \|z\|** | B (95% CI) | **P > \|z\|** | B (95% CI) | **P > \|z\|** | B (95% CI) | **P > \|z\|** | B (95% CI) | **P > \|z\|** |
|  | Model 2 | | Model 3 | | Model 2 | | Model 3 | | Model 2 | | Model 3 | |
| Fixed Effects |  |  |  |  |  |  |  |  |  |  |  |  |
| *Weight perception* *(vs. P. normal weight - M. normal weight)* |  |  |  |  |  |  |  |  |  |  |  |  |
| P. overweight- M. overweight | 1.98 (1.32, 2.64) | <0.001 | 1.47 (0.81, 2.13) | <0.001 | 0.77 (0.36, 18) | <0.001 | 0.53 (0.13, 0.22) | 0.01 | 1.12 (0.72, 1.52) | <0.001 | 0.86 (0.46, 1.26) | <0.001 |
| P. normal - M. overweight | -0.12 (-0.87, 0.63) | 0.761 | -0.42 (-1.16, 0.32) | 0.266 | -0.1 (-0.57, 0.36) | 0.674 | -0.23 (-0.69, 0.90) | 0.322 | 0.09 (-0.36, 0.55) | 0.681 | -0.08 (-0.53, 0.37) | 0.73 |
| P. overweight- M. normal weight | 1.93 (1.14, 2.71) | <0.001 | 1.42 (0.64, 2.20) | <0.001 | 0.62 (0.13, 1.10) | 0.012 | 0.42 (-0.06, 0.94) | 0.082 | 1.33 (0.86, 1.81) | <0.001 | 1.03 (0.55, 1.51) | <0.001 |
| P. underweight- M. normal weight | 1.42 (0.71, 2.13) | <0.001 | 1.57 (0.86, 2.27) | <0.001 | 0.37 (-0.07, 0.81) | 0.104 | 0.34 (-0.08, 0.78) | 0.116 | 1.04 (0.61, 1.47) | <0.001 | 1.21 (0.78, 1.64) | <0.001 |
| P. unsure- M. normal weight | 0.85 (0.35, 1.34) | 0.001 | 0.76 (0.28, 1.25) | 0.002 | 0.43 (0.12, 0.74) | 0.006 | 0.38 (0.08, 0.69) | 0.012 | 0.5 (0.20, 0.81) | 0.001 | 0.47 (0.17, 0.77) | 0.002 |
| P. unsure- M. overweight | -0.06 (-0.79, 0.67) | 0.873 | -0.27 (-1.00, 0.45) | 0.457 | -0.21 (-0.67, 0.24) | 0.353 | -0.25 (-0.70, 0.19) | 0.266 | 0.33 (-0.12, 0.77) | 0.148 | 0.15 (-0.29, 0.59) | 0.499 |
| Random effects |  |  |  |  |  |  |  |  |  |  |  |  |
| Level 3 (School) intercept variance | 0.24 (0.06, 0.98) |  | 0.23 (0.06, 0.95) |  | 0.09 (0.02, 0.37) |  | 0.07 (0.01, 0.34) |  | 0.06 (0.01, 0.38) |  | 0.05 (0.01, 0.38) |  |
| Level 2 (Child) intercept variance | 11.07 (9.73, 12.61) |  | 10.23 (8.97, 11.69) |  | 4.29 (3.77, 4.87) |  | 3.73 (3.25, 4.27) |  | 3.7 (3.22, 4.24) |  | 3.46 (3.00, 3.99) |  |
| Leve l1 (Occasion) intercept variance | 12.72 (11.79, 13.73) |  | 12.34 (11.43, 13.31) |  | 4.89 (4.54, 5.28) |  | 4.72 (4.37, 5.10) |  | 4.84 (4.49, 5.22) |  | 4.81 (4.46, 5.20) |  |
|  | **Males** | | | | | | | | | | | |
|  | **SDQ total difficulties score** | | | | **SDQ externalising difficulties score** | | | | **SDQ internalising difficulties score** | | | |
|  | B (95% CI) | **P > \|z\|** | B (95% CI) | **P > \|z\|** | B (95% CI) | **P > \|z\|** | B (95% CI) | **P > \|z\|** | B (95% CI) | **P > \|z\|** | B (95% CI) | **P > \|z\|** |
|  | Model 2 | | Model 3 | | Model 2 | | Model 3 | | Model 2 | | Model 3 | |
| Fixed Effects |  |  |  |  |  |  |  |  |  |  |  |  |
| *Weight perception* *(vs. P. normal weight - M. normal weight)* |  |  |  |  |  |  |  |  |  |  |  |  |
| P. overweight- M. overweight | 2.12 (1.43, 2.80) | <0.001 | 1.7 (1.00, 2.40) | <0.001 | 0.55 (0.10, 0.99) | 0.016 | 0.52 (0.07, 0.97) | 0.024 | 1.56 (1.16, 1.96) | <0.001 | 1.19 (0.78, 1.60) | <0.001 |
| P. normal - M. overweight | -0.41 (-1.04, 0.23) | 0.209 | -0.67 (-1.30, -0.03) | 0.038 | -0.36 (-0.76, 0.05) | 0.086 | -0.43 (-0.83, -0.02) | 0.040 | 0.04 (-0.33, 0.41) | 0.83 | -0.14 (-0.51, 0.24) | 0.466 |
| P. overweight- M. normal weight | 1.56 (0.38, 2.74) | 0.01 | 1.23 (0.06, 2.41) | 0.039 | 0.77 (0.01, 1.53) | 0.046 | 0.69 (-0.06, 1.45) | 0.070 | 0.69 (-0.01, 1.38) | 0.053 | 0.44 (-0.25, 1.14) | 0.212 |
| P. underweight- M. normal weight | 1.09 (0.55, 1.64) | <0.001 | 1.2 (0.66, 1.74) | 0.001 | 0.46 (0.10, 0.81) | 0.011 | 0.53 (0.14, 0.75) | 0.003 | 0.62 (0.29, 0.94) | <0.001 | 0.65 (0.33, 0.97) | <0.001 |
| P. unsure- M. normal weight | 0.82 (0.34, 1.30) | 0.001 | 0.82 (0.34, 1.29) | 0.389 | 0.41 (0.10, 0.72) | 0.009 | 0.45 (0.14, 0.75) | 0.004 | 0.43 (1.15, 0.71) | 0.003 | 0.38 (0.10, 0.66) | 0.008 |
| P. unsure- M. overweight | 0.55 (-0.18, 1.27) | 0.139 | 0.32 (-0.42, 1.05) | 0.389 | 0.28 (-0.19, 0.74) | 0.243 | 0.3 (-0.17, 0.76) | 0.212 | 0.32 (-0.10, 0.74) | 0.146 | 0.06 (-0.37, 0.49) | 0.779 |
| Random effects |  |  |  |  |  |  |  |  |  |  |  |  |
| Level 3 (School) intercept variance | 0.19 (0.04, 1.04) |  | 0.13 (0.01, 1.12) |  | 0.11 (0.03, 0.42) |  | 0.07 (0.01, 0.40) |  | 0.09 (0.02, 0.34) |  | 0.05 (0.01, 0.33) |  |
| Level 2 (Child) intercept variance | 10.38 (9.17, 11.74) |  | 9.74 (8.57, 11.06) |  | 4.50 (3.99, 5.07) |  | 4.13 (3.65, 4.68) |  | 2.99 (2.61, 3.44) |  | 2.79 (2.41, 3.23) |  |
| Leve l1 (Occasion) intercept variance | 12.75 (11.89, 13.69) |  | 12.62 (11.75, 13.54) |  | 5.19 (4.84, 5.57) |  | 5.14 (4.79, 5.52) |  | 4.65 (4.33, 4.99) |  | 4.64 (4.32, 4.98) |  |

Model 2: adjusted for age and ethnicity

Model 3: adjusted for age, ethnicity, smoking, alcohol consumption, special diets, weight gain anxiety, and physical activity.

**Supplementary Table 3:** The association between weight misperception and probable clinically relevant SDQ total, externalising and internalising symptoms from 11-13 years to 14-16 years (Models 2 and 3)

|  | **Females** | | | | | | | | | | | |
| --- | --- | --- | --- | --- | --- | --- | --- | --- | --- | --- | --- | --- |
|  | **SDQ total difficulties score** | | | | **SDQ externalising difficulties score** | | | | **SDQ internalising difficulties score** | | | |
|  | B (95% CI) | **P > \|z\|** | B (95% CI) | **P > \|z\|** | B (95% CI) | **P > \|z\|** | B (95% CI) | **P > \|z\|** | B (95% CI) | **P > \|z\|** | B (95% CI) | **P > \|z\|** |
|  | Model 2 | | Model 3 | | Model 2 | | Model 3 | | Model 2 | | Model 3 | |
| Fixed Effects |  |  |  |  |  |  |  |  |  |  |  |  |
| *Weight perception* *(vs. P. normal weight - M. normal weight)* |  |  |  |  |  |  |  |  |  |  |  |  |
| P. overweight- M. overweight | 2.74 (1.66, 4.53) | <0.001 | 2.11 (1.26, 3.54) | 0.005 | 1.96 (1.02, 3.72) | 0.041 | 1.53 (0.79, 2.96) | 0.209 | 3.01 (1.73, 5.25) | <0.001 | 2.36 (1.35, 4.12) | 0.002 |
| P. normal - M. overweight | 0.99 (0.50, 1.97) | 0.978 | 0.81 (0.40, 1.62) | 0.551 | 0.75 (0.29, 1.96) | 0.555 | 0.61 (0.23, 1.63) | 0.328 | 1.15 (0.56, 2.38) | 0.700 | 0.97 (0.47, 1.99) | 0.930 |
| P. overweight- M. normal weight | 3.36 (1.85, 6.10) | <0.001 | 2.58 (1.40, 4.74) | 0.002 | 2.02 (0.93, 4.37) | 0.075 | 1.57 (0.71, 3.52) | 0.268 | 4.55 (2.40, 8.60) | <0.001 | 3.26 (1.73, 6.17) | <0.001 |
| P. underweight- M. normal weight | 2.22 (1.26, 3.93) | 0.006 | 2.34 (1.32, 4.17) | 0.004 | 1.06 (0.47, 2.41) | 0.882 | 0.99 (0.43, 2.24) | 0.978 | 2.64 (1.45, 4.82) | 0.001 | 3.36 (1.84, 6.15) | <0.001 |
| P. unsure- M. normal weight | 1.80 (1.17, 2.78) | 0.008 | 1.67 (1.08, 2.58) | 0.022 | 1.48 (0.84, 2.59) | 0.174 | 1.38 (0.78, 2.42) | 0.266 | 1.76 (1.09, 2.82) | 0.019 | 1.68 (1.05, 2.69) | 0.029 |
| P. unsure- M. overweight | 1.32 (0.70, 2.48) | 0.387 | 1.20 (0.63, 2.27) | 0.573 | 1.04 (0.44, 2.46) | 0.927 | 1.05 (0.44, 2.47) | 0.914 | 1.86 (0.97, 3.55) | 0.062 | 1.51 (0.79, 2.89) | 0.208 |
| Random effects |  |  |  |  |  |  |  |  |  |  |  |  |
| Level 3 (school) Intercept | 0.06 (0.01, 0.59) |  | 0.06 (0.01, 0.69) |  | 0.00 (0.00, 0.00) |  | 0.00 (0.00, 0.00) |  | 0.15 (0.03, 0.64) |  | 0.13 (0.03, 0.60) |  |
| Level 3 (school)> Level 2 (individual) Intercept | 2.64 (1.71, 4.06) |  | 2.42 (1.54, 3.80) |  | 3.48 (1.96, 6.20) |  | 2.81 (1.52, 5.21) |  | 3.21 (2.08, 4.93) |  | 2.74 (1.75, 4.33) |  |
|  | Males | | | | | | | | | | | |
|  | **SDQ total difficulties score** | | | | **SDQ externalising difficulties score** | | | | **SDQ internalising difficulties score** | | | |
|  | B (95% CI) | **P > \|z\|** | B (95% CI) | **P > \|z\|** | B (95% CI) | **P > \|z\|** | B (95% CI) | **P > \|z\|** | B (95% CI) | **P > \|z\|** | B (95% CI) | **P > \|z\|** |
|  | Model 2 | | Model 3 | | Model 2 | | Model 3 | | Model 2 | | Model 3 | |
| Fixed Effects |  |  |  |  |  |  |  |  |  |  |  |  |
| *Weight perception* *(vs. P. normal weight - M. normal weight)* |  |  |  |  |  |  |  |  |  |  |  |  |
| P. overweight- M. overweight | 3.15 (1.81, 5.45) | <0.001 | 2.41 (1.35, 4.29) | 0.003 | 1.20 (0.61, 2.37) | 0.591 | 1.11 (0.55, 2.27) | 0.768 | 5.30 (2.82, 9.95) | <0.001 | 3.71 (1.92, 7.14) | <0.001 |
| P. normal - M. overweight | 0.76 (0.39, 1.51) | 0.440 | 0.64 (0.32, 1.28) | 0.212 | 0.57 (0.25, 1.29) | 0.178 | 0.51 (0.22, 1.17) | 0.114 | 1.00 (0.46, 2.19) | 0.999 | 0.76 (0.34, 1.68) | 0.490 |
| P. overweight- M. normal weight | 1.71 (0.56, 5.16) | 0.342 | 1.45 (0.48, 4.39) | 0.508 | 2.84 (0.96, 8.44) | 0.060 | 2.67 (0.90, 7.91) | 0.076 | 4.15 (1.38, 12.50) | 0.011 | 3.12 (1.03, 9.44) | 0.044 |
| P. underweight- M. normal weight | 2.04 (1.25, 3.34) | 0.004 | 2.19 (1.34, 3.57) | 0.002 | 1.94 (1.14, 3.28) | 0.013 | 2.09 (1.24, 3.54) | 0.006 | 2.55 (1.42, 4.60) | 0.002 | 2.67 (1.48, 4.81) | <0.001 |
| P. unsure- M. normal weight | 1.62 (1.03, 2.57) | 0.038 | 1.57 (0.99, 2.49) | 0.057 | 1.23 (0.73, 2.09) | 0.429 | 1.25 (.73, 2.12) | 0.418 | 1.36 (0.77, 2.41) | 0.29 | 1.28 (0.72, 2.28) | 0.402 |
| P. unsure- M. overweight | 1.47 (0.75, 2.90) | 0.267 | 1.26 (0.63, 2.53) | 0.506 | 1.27 (0.58, 2.77) | 0.554 | 1.29 (0.58, 2.86) | 0.536 | 0.99 (0.41, 2.74) | 0.992 | 0.77 (0.31, 1.92) | 0.581 |
| Random effects |  |  |  |  |  |  |  |  |  |  |  |  |
| Level 3 (school) Intercept | 0.09 (0.01, 0.79) |  | 0.08 (0.00, 0.87) |  | 0.22 (0.07, 0.74) |  | 0.19 (0.05, 0.71) |  | 0.00 (0.00, 0.00) |  | 0.00 (0.00, 0.00) |  |
| Level 3 (school)> Level 2 (individual) Intercept | 2.81 (1.79, 4.39) |  | 2.61 (1.63, 4.17) |  | 3.04 (1.81, 5.10) |  | 2.74 (1.60, 4.69) |  | 3.15 (1.86, 5.34) |  | 2.89 (1.64, 5.11) |  |

Model 2: adjusted for age and ethnicity

Model 3: adjusted for age, ethnicity, smoking, alcohol consumption, special diets, weight gain anxiety, and physical activity.
